# Do Asian elephants plan for mutually-exclusive outcomes?

**DOI:** 10.1007/s10071-025-02009-1

**Published:** 2025-11-12

**Authors:** Sydney F. Hope, Sangpa Dittakul, Marnoch Yindee, Taweepoke Angkawanish, Joshua M. Plotnik

**Affiliations:** 1 Department of Psychology, Hunter College, City University of New York, New York, NY USA; 2 Golden Triangle Asian Elephant Foundation, Chiang Saen, Chiang Rai, Thailand; 3 Akkhraratchakumari Veterinary College, Walailak University, Nakhon Si Thammarat, Thailand; 4 National Elephant Institute, Forest Industry Organization, Lampang, Thailand; 5 Psychology Ph.D. Program, CUNY Graduate Center, City University of New York, New York, NY USA

**Keywords:** Future planning, Mutually-exclusive outcomes, Collective intelligence, Social context, *Elephas maximus*, Asian elephant

## Abstract

The ability to prepare for mutually-exclusive outcomes is critical for future planning. Recently, the thought that this ability may be unique to humans has been questioned. Even if non-human animals cannot *individually* plan for mutually-exclusive outcomes, groups of individuals may be able to coordinate their behavior to do so *collectively*. Here, we tested 12 Asian elephants (*Elephas maximus*), both individually and in pairs, using a forked tube task—adapted from one used with children and non-human apes—where a food reward is dropped down a tube and exits from one of two openings. The consistent, simultaneous covering of both openings to obtain the reward is evidence of an understanding of mutually-exclusive outcomes. One elephant—Nammei—learned to manipulate her trunk in a scooping motion to autonomously cover both openings, and then performed this behavior relatively consistently to successfully obtain the food reward at a rate significantly greater than chance (61.5%). In addition, pairs of elephants obtained the food reward at a rate significantly greater than that at which individuals could do by chance (i.e., either elephant ate the food in 60.1% of pair trials). However, Nammei eventually reverted to covering only one opening, and pairs did not achieve complete coordination—in fact, both openings were covered in only 35.0% of pair trials. Therefore, our results fall short of providing compelling evidence for either individual or collective planning for mutually-exclusive outcomes in elephants. However, the interesting behaviors that we observed suggest that this is a promising area for future research.

**Supplementary Information:**

The online version contains supplementary material available at 10.1007/s10071-025-02009-1.

## Introduction

The ability to plan for the future is an aspect of cognition that is integral to human evolution, but less is known about this ability in non-human animals. Although there is debate as to whether non-human animals are capable of mentally representing and deliberately planning for the future (Suddendorf and Corballis 2007; Redshaw and Bulley 2018), there is some evidence that they do prepare for future events. For example, great apes select (Mulcahy and Call 2006; Osvath and Osvath 2008) and produce (Bräuer and Call 2015) appropriate tools and save them for future use. Corvids demonstrate that they can plan for future hunger by preferentially caching food in a location in which they have learned they would be food-deprived the next morning (Raby et al. 2007) and that they continue to cache food based on their anticipated future motivational state even if it contradicts their current state (Correia et al. 2007; Cheke and Clayton 2012). The ability to imagine future scenarios is also thought to be linked to the ability to recall mental images of past events, or episodic memory (Cheke and Clayton 2010; Clayton 2017)—together referred to as ‘mental time travel’. Therefore, investigating whether non-human animals can plan for the future is an important step towards understanding whether they can recollect events in the past or imagine events in the future, and if they experience mental time travel similarly to humans (Zentall 2013; Clayton 2017).

Not only can humans plan for specific future events, but we can also imagine mulitple possible scenarios and develop contingency plans. This ability to plan for multiple possibilities when the outcome of an event is uncertain—and being consciously aware that each scenario is a different representation of the future—may be unique to humans (Redshaw and Bulley 2018). Redshaw and Suddendorf (2016) investigated the ontogeny and evolution of this cognitive ability using an elegant forked tube paradigm, where an experimenter dropped a reward down a single tube and it would exit from one of two bottom openings. They first allowed subjects—human children and non-human great apes—to watch how the forked tube worked, and then subjects were given the opportunity to catch the reward. Researchers found that 4 year-old children immediately and consistently used both hands to cover both openings, suggesting that they used insight to mentally, and then physically, prepare for the two mutually-exclusive outcomes. However, 2 year-old children and great apes only covered one opening and tried to predict from which side the food would fall, suggesting that they prepared for only one possible future outcome. Subsequent studies have provided mixed evidence for whether great apes and other primates can prepare for both outcomes in this forked tube task (Suddendorf et al. 2017, 2020; Lambert and Osvath 2018; Warren et al., unpublished data; also see Engelmann et al. 2021, 2023 for different methods). However, knowledge of the ability to plan for mutually-exclusive outcomes in non-primates is limited.

Even if non-human animals cannot plan for mutually-exclusive future events individually, it is possible that ‘emergent planning’ may occur when they act collectively. When animals make decisions as part of a collective, new reasoning-like intelligence—or ‘collective intelligence’—may arise (Couzin 2009; Theiner et al. 2010; Whiten et al. 2022; but see Rupert 2004 and Adams and Aizawa 2010 for critiques of the hypothesis that cognition can extend beyond the individual). In its simplest form, collective intelligence can be defined as when the collective performs better than individuals working separately (Pratt 2009; Green 2015). For example, groups may find solutions to problems that individuals alone cannot (Krause et al. 2010). However, the cognitive mechanism underlying the intelligence of a collective is not always the same. In its most cognitively complex form, collective intelligence can arise through explicit organizational decisions (Theiner et al. 2010) and consensus decision-making (Conradt and Roper 2005). It can also arise in the absence of individual understanding or reasoning (Pratt 2009; Theiner et al. 2010). Indeed, in many cases, collective action is self-organized with no central control and, instead, many simple local interactions form larger patterns (Camazine et al. 2020). For example, collective movements of large groups of animals are often self-organized and dependent on local interactions between few individuals (Petit and Bon 2010). Similarly, it is possible that the anticipation of future scenarios may emerge within a collective, even when individuals cannot anticipate all outcomes. For example, observational studies have shown that, when hunting, groups of wild chimpanzees collectively block their prey’s multiple escape routes (Boesch 2002). Thus, although each chimpanzee only blocks one escape route, all routes are ultimately blocked—accounting for all future possibilities of the prey escaping in a particular direction. Interestingly, whether or not chimpanzees cognitively understand that they are collectively accounting for all outcomes (Stanford et al. 1994) does not change whether this behavior constitutes collective intelligence. In other words, even a more parsimonious cognitive explanation for the behavior—that the individual simply covers an escape route that is not already occupied without any understanding of what conspecifics are doing—may represent collective intelligence, as this still maximizes potential success (Pratt 2009).

One animal that may exhibit future planning for mutually-exclusive outcomes both individually and collectively is the Asian elephant (*Elephas maximus*). Asian elephants are large-brained mammals (Shoshani et al. 2006; Hart et al. 2008; Herculano-Houzel et al. 2014) that exhibit complex cognition, such as innovative (Greco et al. 2013; Barrett and Benson-Amram 2021; Jacobson et al. 2022, 2023) and insightful (Foerder et al. 2011) problem-solving, tool-use (Chevalier-Skolnikoff and Liska 1993; Hart and Hart 1994; Hart et al. 2001; Foerder et al. 2011; Barrett and Benson-Amram 2020), and body/self-awareness (Plotnik et al. 2006, 2010; Dale and Plotnik 2017). Although little is known about whether elephants experience ‘mental time-travel’, there is some evidence that elephants have strong long-term memories (reviewed in Hope et al. 2024) and can plan for the future. For example, there is anecdotal evidence that captive Asian elephants, who often wear bells secured around their necks to alert their handlers of their location in the forest, stuff them with mud to possibly prevent detection later in the night (Williams 1950). Importantly, elephants may also be able to collectively plan for the future. Elephants are social animals, live in fission-fusion societies (de Silva et al. 2011; de Silva and Wittemyer 2012) and exhibit complex social behaviors, such as allomothering (Lee 1987; Schulte 2000; Bates et al. 2008; Vidya 2014)—suggesting that they may make decisions collectively and cooperatively. Indeed, Asian elephants can work together to solve problems. In a task where two elephants each needed to pull one end of a single rope threaded through and around a food-baited, out-of-reach table in order to move the table towards them, elephants waited to pull until partners arrived up to 45 s later (Plotnik et al. 2011). In a separate study, when elephants were free to choose their own partner in a similar rope-pulling task, they mitigated competition in order to maximize cooperation (Li et al. 2021). However, when the food was not shareable and one individual could monopolize it after pulling cooperatively, cooperation quickly collapsed regardless of rank relationships (Li et al. 2021). Together, this may suggest that elephants plan for future outcomes depending on their social surroundings, and may plan accordingly depending on whether there are potential future benefits. However, whether Asian elephants may be able to plan for mutually-exclusive future events either individually or collectively has never been experimentally tested.

Here, we investigated whether Asian elephants can plan for mutually-exclusive future outcomes individually and/or collectively using a simple forked tube paradigm, similar to that of Redshaw and Suddendorf (2016). We predicted that, if individual elephants can plan for mutually-exclusive future events, they would spontaneously learn to cover both sides of the forked tube by using a ‘scooping’ motion with their trunk, and then continue this behavior consistently. This would show that they understood that there was an equal probability of the reward coming out of either opening. Unlike Redshaw and Suddendorf (2016) who used ‘demonstration trials’ to first show subjects how the reward could fall from the forked tube and then tested whether subjects covered both sides using insight on the first trial, we decided not to test this in elephants because they likely prioritize olfactory information in foraging tasks over vision (Shoshani et al. 2006; Jacobson and Plotnik 2020); we were concerned that visually-biased demonstration trials would not be ecologically relevant for elephants. Instead, we decided to test whether elephants learned to use the strategy of covering both openings after they had experience with the touch, smell, and sounds of the experimental trials, and expected that, if they were using insight, they would quickly produce the solution of covering both openings after experience with only a few trials. Because we expected that an elephant’s single appendage might occasionally bend in such a way that it covered both openings by chance, we were particularly interested in whether elephants consistently performed this behavior. As an additional way to investigate how elephants might understand the contingencies of the forked tube, we also investigated whether elephants always covered the same side of the tube or if they switched between different sides. Switching sides would suggest that they were actively trying to predict the side from which the reward would fall. In contrast, elephants might continuously cover the same side for different reasons. First, consistently covering one side may suggest that the elephant has given up on attempting to predict a side. Then, as they cover a particular side more often, they may continue to choose it because it is now the side from which they believe they were most often rewarded (Redshaw and Suddendorf 2020). Alternatively, if elephants understand that there is an equal possibility of the food coming out of either side, but they cannot or do not want to cover both sides, they may choose to cover one side consistently because it is a strategy that guarantees 50% success.

To determine whether emergent planning for mutually-exclusive outcomes may arise when elephants act collectively, even if they cannot do so individually, we also tested the forked tube paradigm with elephant pairs. We predicted that, if elephants collectively plan for mutually-exclusive future events, pairs would collectively account for the reward coming out of either side of the tube. With perfect coordination, this would result in both sides being covered—and food being eaten by an elephant—in 100% of the trials (and each individual receiving a reward 50% of the time). However, even if the food reward was eaten at a rate > 50% (i.e., the maximum that an individual could achieve if only covering one side), it would still meet the most basic definition of collective intelligence—which is that the collective performs better than the individual (Pratt 2009; Green 2015). Alternatively, because the reward was not shareable, we thought that competition might counteract collective behavior in two ways. First, if individual elephants do not understand that there is an equal probability of food falling from either side, then the non-shareable food reward may reduce the likelihood of them acting as a collective (Li et al. 2021). For example, if elephants try to predict from which side the food will fall, then they may compete to cover the same side of the tube—which would reduce collective success. In contrast, competition could foster innovation (Brosnan and Hopper 2014). If this is the case, we would expect that individual elephants may begin covering both sides (i.e., ‘scooping’) when they are in pairs to monopolize the food.

## Methods

### Subjects

We worked with 12 Asian elephants (11 females and 1 male, ages 4–58 years) at the National Elephant Institute (NEI) in Lampang, Thailand from August – October 2023. The NEI is home to > 80 elephants and has the largest elephant hospital in Thailand; elephant health is monitored by ~ 10 full-time veterinarians. Each elephant also has their own mahout, or elephant caretaker, who is responsible for daily husbandry and training. No elephant had ever had the opportunity to previously interact with an apparatus similar to the one that we used in this study. Several of the elephants had participated in Plotnik et al. (2011) 17 years ago, and two elephants (Wandee and Sanlan) participated in Schmitt (2025) shortly before or concurrently with this study but this was the only experience any of them had had with cognitive research, and the studies tested cooperation and inhibitory control, respectively, using completely unrelated apparatuses. Elephants in this study were tested both individually and in pairs. Six unique pairings were formed based on the advice of mahouts and veterinarians to ensure the probability of antagonistic interactions was minimized. Demographic and pair information can be found in Table 1.

**Table 1:** Information about elephant demographics, pairing, number of trials, and covering both sides/openings of the tube

Name	Sex	Age (years)	Paired with	Type of trial to start	Number of trials individually	Number of trials in pairs	Covered both at least once?	Trials covered both individually	Trials covered both in pairs
Nammei	F	16	Pachee	Individual	299	252	Y	89	16
Pachee	F	15	Nammei	Individual	181^a^	252	Y	1	0
Phumpuong	F	41	Khod	Individual	180	180	Y	8	1^b^
Khod	F	41	Phumpuong	Individual	177	180	Y	3	0
Alina	F	19	Malini	Pair	180	181^a^	Y	1	0
Malini	F	19	Alina	Pair	180	181^a^	N	0	0
Lawan	F	44	Wandee	Pair	180	180	Y	0	2
Wandee	F	58	Lawan	Pair	180	180	Y	2	0
Malee	F	9	Sanlan	Pair	180	180	Y	1	0
Sanlan	M	6	Malee	Pair	180	180	N	0	0
Somsri	F	10	Baiboon	Individual	180	180	N	0	0
Baiboon	F	5	Somsri	Individual	180	180	N	0	0

The type of trial with which each elephant began testing (individual or pair), the number of total trials they received when tested individually and when paired, whether or not they covered both sides/openings of the tube at least once (Y or N), and the number of individual and pair trials in which they covered both sides are reported

^a^Two extra trials were run accidentally during the study; Pachee received one extra test trial as an individual, and Alina and Malini received one extra test trial as a pair

^b^In this single trial, Phumpuong put her trunk under the left side but then quickly switched to the right side as the food dropped. This was coded as ‘cover both’ for analyses, but she did not scoop her trunk. This was the only instance in which an elephant performed a behavior like this

### Experimental apparatuses and set-up

**Fig. 1:**
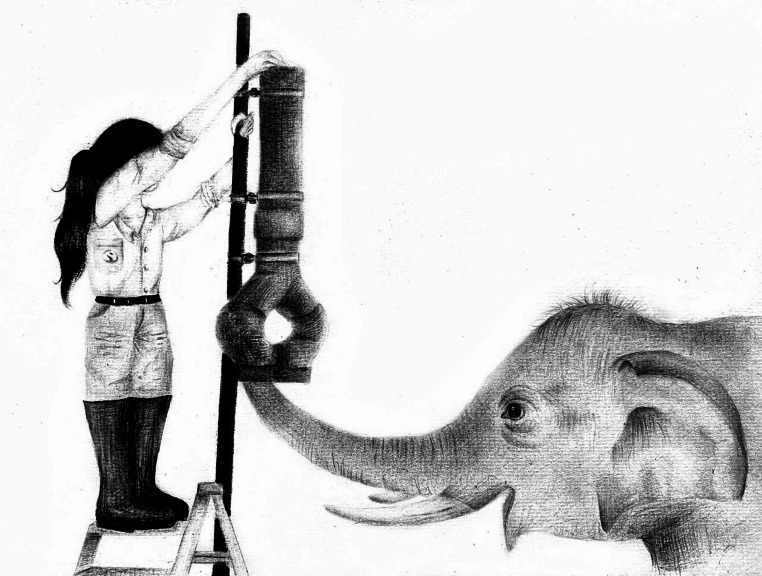
The forked-tube set-up, with experimenter preparing to drop a food reward down one side of the tube. The elephant has already chosen a side. Sketch by S.D., inspired by a photo taken by Mathias Dezetter. Sketch is not to scale

We adapted our methods from Redshaw and Suddendorf (2016). We used two different apparatuses: a forked tube for testing (Fig. 1) and a straight tube for training. We made the forked tube by connecting a 50 cm-long piece of PVC pipe with a 9 cm diameter to a forked Y tube pipe fitting. We then connected a 90-degree curved pipe fitting to each side of the forked pipe fitting, followed by a 45-degree curved pipe fitting. The length of the entire apparatus was 1 m and the pipe fittings were oriented in such a way that the distance from one end of the bottom opening to the other end was 24 cm. We also secured a plastic sheet separator in the center of the top portion of the forked tube. The plastic separator started ~ 3 cm from the top opening and extended into the forked tube pipe fitting, so that if food was dropped slightly to the left or slightly to the right, it was certain to fall down the respective side of the apparatus. This separation was not visible to the elephant. The straight tube that was used for training was a straight PVC pipe that was 1 m long and 9 cm in diameter, with a straight pipe fitting to mimic the appearance and texture of the curved pipe fittings of the forked tube.

During testing and training, we secured the appropriate tube to a stationary pole that was placed on the side of the fence opposite to the elephant. Underneath the tube, we attached a 122 × 65 cm ramp made of plastic, which was positioned in such a way that, when the food fell out of either opening, it fell onto the ramp and away from the elephant, into a large trough, making it inaccessible to the elephant. We used a step-ladder that we placed inside the trough to reach the top opening to drop the food. All trials were video recorded using Sony (models FDR-AX100, HDR-CX405) and/or GoPro (models HERO7 White, HERO8 Black) cameras.

### Experimental protocol

#### Straight tube shaping

Because elephants were not familiar with catching objects out of a tube, we performed a three-step shaping process using the straight tube. First, we inserted a long stick with food attached to the end inside of the tube so that the food was hanging out. For all pairs except Malee and Sanlan, the food reward was a small piece of apple. Because Malee was not motivated by apples, we used small pieces of sugarcane for her and her partner (Sanlan). Next, once the elephant consistently ate the food off of the stick, we began dropping food down the tube at the same time that the elephant was removing the food from the stick. Lastly, once the elephant began consistently catching the food being dropped, we removed the stick. During shaping, elephants’ mahouts (handlers) and experimenters used the following commands to teach elephants to catch the food: *jap* (“touch”), *maa* (“come”), *bon* (“lift trunk up”), *luum* (“put trunk down”), and *yaa* (“stop”). In addition, they could gesture underneath the tube as needed. However, we paid special attention to not guide the elephant’s trunk inside the tube. This was especially important because we did not want to bias the elephant’s behavior to put their trunk inside the tube, because any such bias could affect how they reacted to the forked tube later. Once the elephant began consistently catching the food without the stick, we stopped giving commands and gesturing. Once the elephant caught the food three consecutive times without the stick or any commands or gestures, we moved to *straight tube training*. All elephants completed shaping within 3 days (maximum of 1 h per day).

#### Straight tube training

To ensure that elephants were able to consistently catch food from a tube, they underwent straight tube training. One experimenter (S.D.) first held one piece of food at the top of the straight tube for 5 s and then dropped it (regardless of whether the elephant’s trunk was underneath or not). If the elephant did not catch the food, it rolled onto the ramp and out of reach. Each time the food was dropped was considered one trial, and trials continued without interruption. If necessary, elephants’ mahouts (handlers) and experimenters were allowed to use the same commands as during *straight tube shaping*, but were not allowed to gesture. Elephants passed training when they caught the food in five consecutive trials. A ‘catch’ was defined as any time that the elephant ate the food, which included trials in which they either caught the food in their trunk or when they used their trunk to knock the food onto their side of the fence. If an elephant was uninterested (i.e., did not try to catch the food) during 12 consecutive trials, we gave them three pieces of apple underneath the tube to increase motivation. If the elephant remained uninterested, we stopped training and continued after taking a break. All elephants passed training within a single day (range of 5-sec trials needed to complete training: 5–102), and then moved to the forked tube testing. If the first time that an elephant experienced forked tube testing was on a different day than when they passed straight tube training, we proceeded to the forked tube only after repeating straight-tube training until the elephant caught the food three consecutive times.

#### Forked tube testing

Forked tube testing was performed in sets of 12 trials each (i.e., one trial = one instance of food dropping into the tube). However, a set was still considered a ‘full set’ if an elephant covered one or both ends of the forked tube for at least ten consecutive trials without losing interest. Forked tube testing was carried out on both individuals and pairs. Three pairs first completed three full sets of forked tube testing individually before participating in paired testing. The other three pairs first completed three full sets of forked tube testing in pairs, before being tested individually. After this, pairs alternated between three full sets of individual or pair testing until they completed 15 sets in each category. Because Nammei was the only elephant who covered both sides consistently and we wanted to investigate this behavior further, we conducted ten additional sets with her individually and six additional sets with her paired with her partner, Pachee. The number of trials for each elephant and pair are listed in Table 1. During all trials, the elephants’ mahouts (handlers) were asked to stand away from the elephant and only give the following commands in the following situations: *maa* (“come”; if the elephant walked away from or was not oriented toward the apparatus), *bon* (“lift trunk up”; to tell the elephant to interact with the apparatus), *luum* (“move trunk down”; to tell the elephant to put their trunk down if they tried to grab the food from the experimenter’s hand at the top of the tube), and *yaa* (“no”; if elephants tried to grab the food from the experimenter or pull the pole/tube).

We expected that elephants would need to experience (1) the physical feeling that there were two openings, (2) smelling the food at both openings, and (3) hearing the food drop to the ramp and roll away when they did not cover the correct opening. We used three manipulations to attempt to demonstrate to the elephant that food could come out of either side. First, when we installed the forked tube, the elephant was allowed to inspect the forked tube for at least 30 s before the first trial began. Second, we rubbed a piece of food (i.e., apple or sugar cane) on the bottom of both openings so the elephant could have the odor cue to understand that food may come out of either side. Third, the experimenter gave two pieces of reward food (one directly under each opening of the forked tube) three times in a row to the elephant before trials in a given set began. Food was given in this manner between sets as needed to maintain the elephants’ interest, but never during a set. Food was rubbed on the bottom of the tubes as needed between trials to maintain interest. If elephants lost interest (i.e., walked away from the apparatus or did not respond to the mahout’s calls to engage with the experiment) before completing ten consecutive trials, we excluded those trials and gave elephants supplementary pieces of food underneath the forked tube. If the elephant regained interest, we restarted and attempted to complete the set again, but if they did not, we stopped experimentation for that day.

Each trial began when one experimenter (S.D.) held the food at the top of the tube, and the elephant was given 5 s to decide where to put their trunk. After 5 s, if the elephant had chosen a side, the other experimenter (S.F.H.) tapped S.D. on the shoulder corresponding to the side in which the food should be dropped (Supplementary Video 1). An elephant was defined as ‘choosing a side’ when their trunk was completely covering either one or both holes. The order of dropping was predetermined before each trial and pseudorandomized so that the food never dropped out of the same side more than three times consecutively. If, after 5 s, the elephant had not chosen a side, S.F.H. waited until they had, and then tapped S.D.’s shoulder. Trials followed immediately after the previous trial had ended, until the set had been completed. If the food got stuck in the tube (13 trials total across all elephants), we excluded that trial and repeated it at the end of the set. In five trials (total, across all elephants), S.F.H. tapped S.D.’s shoulder on a certain side, but the food dropped from the opposite. In all instances we corrected for this in real-time, so that these sets maintained a 1:1 ratio of food dropping from the left and right sides.

Trials with paired elephants followed the same protocol as the individual trials. Only one apparatus was used for each pair. Elephants stood side by side and the apparatus was placed directly between them, ensuring that each elephant’s trunk could reach both openings. We changed which side elephants stood on so that elephants were never tested on the same side more than twice in a row on the same day, and each elephant was tested an approximately equal number of times on each side. We dropped the food when either one or both elephants had chosen a side (i.e., their trunk was completely covering either one or both openings).

### Behavioral coding

We watched all videos to verify the side from which food dropped (left or right) and to determine which side(s) the elephant covered (left, right, or both). All sides were coded from the experimenter’s perspective. The side(s) that the elephant covered was coded when S.F.H. tapped S.D. on the shoulder, as this coincided with the time that the elephant chose a side (i.e., S.F.H. tapped S.D. on the shoulder once the elephant chose a side). Because the elephant was required to make a choice before the food was dropped, it was unlikely the elephant could use any external cues (e.g., the sound or smell of the food dropping) to predict from which side the food would come out. In pair trials, because only one elephant needed to cover a side in order for the food to be dropped, it was possible that one of the elephants could be scored as covering ‘none’.

‘Covering both’ was defined as when the trunk was fully underneath both openings. The trunk did not need to touch both openings nor did the elephant need to successfully catch the food to be coded as ‘both’, but rather, the surface area of the trunk needed to be fully under both openings so that the food could plausibly be caught in the trunk if it fell out of either opening. In every instance, when this ‘covering both’ behavior occurred, an elephant covered both openings by putting the tip of their trunk into one opening, and ‘scooping’ (i.e., creating a U-shape or cradle with the rest of their trunk) underneath the other opening; we noted to which side the tip of their trunk was directed and to which side they scooped in every instance. The direction in which elephants scooped was always consistent within individuals (Supplementary Material 1, Table S1).

The ultimate measure of success in this experiment was how often the elephant obtained food. In every trial, we noted whether the elephant ate the food (yes or no). This was especially important for when elephants covered both sides. Because it was easier for elephants to catch the food if it dropped from the opening towards which the tip of their trunk was directed rather than the opening under which their trunk scooped, even if an elephant covered both openings, it did not necessarily mean that they succeeded at catching the food. In addition, occasionally, due to malfunction of the ramp, an elephant covered the incorrect side but was still able to eat the food reward.

Furthermore, to investigate whether elephants were trying to predict from which side the food would come out or if they remained consistent with their side choice across trials, we also counted/scored the number of times that, within a set, an elephant changed their choice of side. For example, an elephant who put their trunk under the left side for all 12 trials would receive a ‘0’, while an elephant who chose left five times, both one time, and right six times, would receive a ‘2’ because they switched their choice two times (i.e., from left to both, and then from both to right). In pairs, if one elephant did not cover a side (i.e., ‘none’), this trial was excluded in the calculation of number of switches. We also noted instances (yes or no) where the elephant moved their trunk back and forth between both sides as the food was being dropped (i.e., between the time when S.F.H. tapped S.D.’s shoulder and when the food fell from the opening), which could suggest that they treated both openings as independent but equally valuable outcomes (Redshaw and Suddendorf 2020), but did not understand that the outcomes were mutually exclusive. However, this behavior occurred in only a single trial (i.e., elephants almost always made a choice which opening to cover before the food was dropped), and so we did not analyze it further.

In addition to coding all of the above behavioral endpoints for every individual elephant whether the testing was individual or in pairs, we also considered other variables for trials conducted in pairs. To investigate whether elephants may collectively succeed at covering both sides of the forked tube while in pairs, we noted whether both openings were covered (i.e., either by each elephant covering one opening or one elephant covering both openings) and whether the food was eaten by either elephant. We noted whether both elephants attempted to cover the same opening (Supplementary Material 1, Fig. S1), which could indicate that they were trying to predict or compete for an opening that they thought was the ‘correct’ one, and thus may not understand that there was an equal probability of the food coming out of either opening. Furthermore, to investigate the strategy by which elephants may have coordinated their behavior, we noted if either or both elephants covered the side that was farthest from where they were standing (including if they covered both sides). This behavior may indicate competition, where one elephant attempts to block their partner’s access to the tube, or could be a social behavior, signaling trust when positioning their trunk close to their partner.

We also analyzed the behavior of the one elephant who consistently covered both sides (Nammei) when with her partner (Pachee) in more detail to determine whether Nammei might either (1) revert to covering one side while with her partner, which would allow her partner an opportunity to eat the food or (2) attempt to monopolize the forked tube. In all of Nammei’s pair trials, we coded whether Pachee was interacting with the tube or not, where ‘interacting’ was defined as putting her trunk under one or both openings and/or touching any part of the forked tube. In all of Nammei’s pair trials subsequent to the first trial in which she covered both sides, we also noted whether Nammei attempted to cover both sides but was unable to because Pachee pushed her trunk out of the way (Supplementary Material 1, Fig. S2).

The second author (S.D.) coded all trials for: the side from which the food dropped, the side the elephant covered (left, right, both, or none [only in pairs]), and whether the elephant ate the food. From these raw data, we were also able to record whether pairs of elephants covered the same or opposite sides, and calculate the number of switches. To assess inter-rater reliability for the raw data, another independent observer coded 20.1% of trials (*N* = 922 trials). We chose these trials by randomly selecting 20.1% of each subject’s individual and pair sets, ensuring equal distribution among individuals and between individual and pair trials. In addition, because only one elephant (Nammei) consistently covered both sides of the tube and we wanted to be certain of these codes, the first author (S.F.H.) also independently coded all of Nammei’s sets (*N* = 551 trials). Furthermore, S.F.H. coded: (1) for all of Nammei's pair trials, whether Pachee was interacting with the tube and (2) for all of Nammei’s pair trials subsequent to the first time she covered both sides, whether Nammei attempted to cover both sides but was unable to because Pachee pushed her trunk out of the way. S.D. coded these two behaviors in 23.8% of trials (*N* = 60 out of all 252 pair trials) and 31.2% of trials (*N* = 48 out of 154 pair trials subsequent to the first time Nammei covered both), respectively, to assess inter-rater reliability.

### Statistical analyses

All statistical analyses were conducted using R statistical software version 4.3.1 (R Core Team 2023). To determine whether elephants were successful (i.e., ate the food) at rates greater than chance (50%), we used one-sided tests of proportions. To determine whether Nammei differed in the frequency at which she covered both sides of the tube depending on whether she was tested individually or in a pair, we used a general linear mixed effects model with a binomial distribution, with whether she covered both sides (yes or no) as the dependent variable and whether the trial was individual or in a pair as the independent variable. We also included trial number (continuous numbering) as a random slope and date as a random intercept to account for temporal autocorrelation. Lastly, to determine whether the number of times that elephants switched sides within a set changed over time or differed when tested individually or in pairs, we used a general linear mixed model with a Poisson distribution for count data. The number of times that elephants switched sides within a set was the dependent variable and the overall set number and whether the set was conducted individually or in a pair were the two independent variables. For this last analysis, the extra sets conducted with Nammei and Pachee were excluded, so that only the 15 individual and 15 pair sets of all elephants could be compared. Because there were multiple sets with the same elephant, we included elephant ID as a random effect. All mixed effects models were run using the *lme4* package (Bates et al. 2015), and test statistics and *p*-values were calculated using the *Anova* function of the *car* package (Fox and Weisberg 2019). Finally, we calculated Cohen’s Kappa using the *irr* package (Gamer et al. 2019) to determine inter-rater reliability for all variables.

## Results

### Covering both sides of the tube individually

**Fig. 2:**
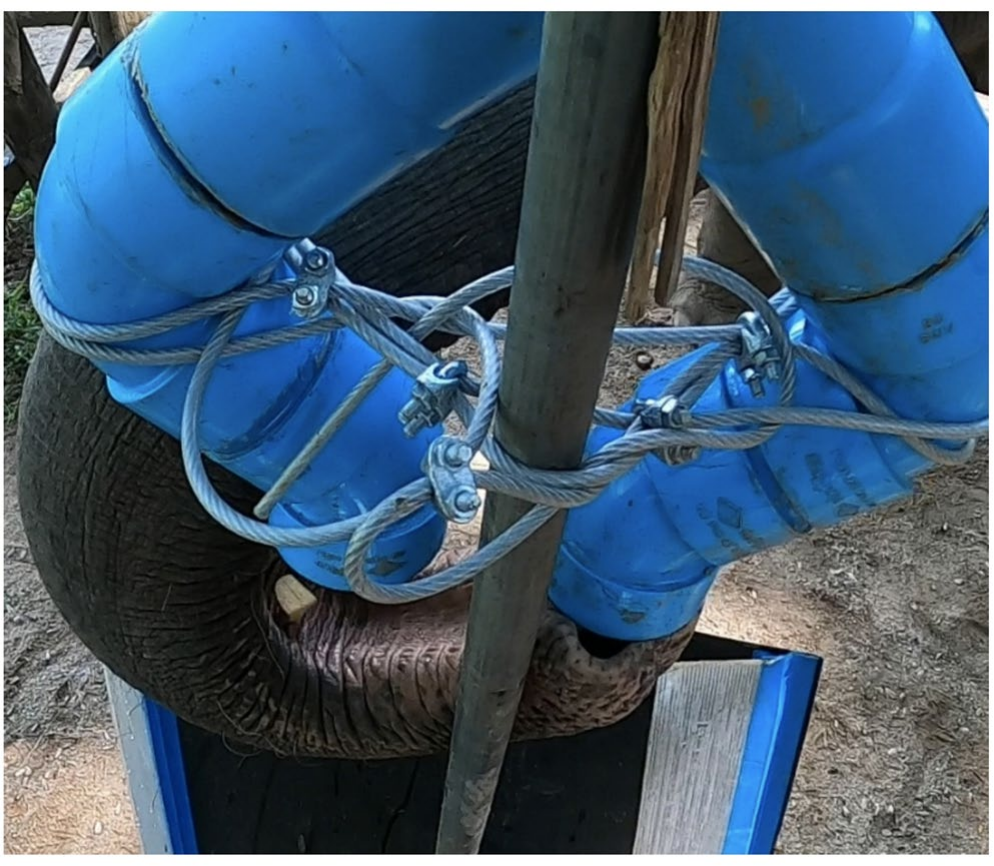
Nammei performing the ‘scoop’ to cover both ends of the tube. The tip of her trunk is in the right end and her trunk scoops to the left to cover the left end (the photo and left/right sides mentioned here are from the experimenters’ perspective). Notice that she caught a piece of apple, which fell out of the left side, in the scoop/elbow of her trunk. Photo is a still image captured and cropped from a video taken with a GoPro HERO8 which was chest mounted on the experimenter

**Fig. 3:**
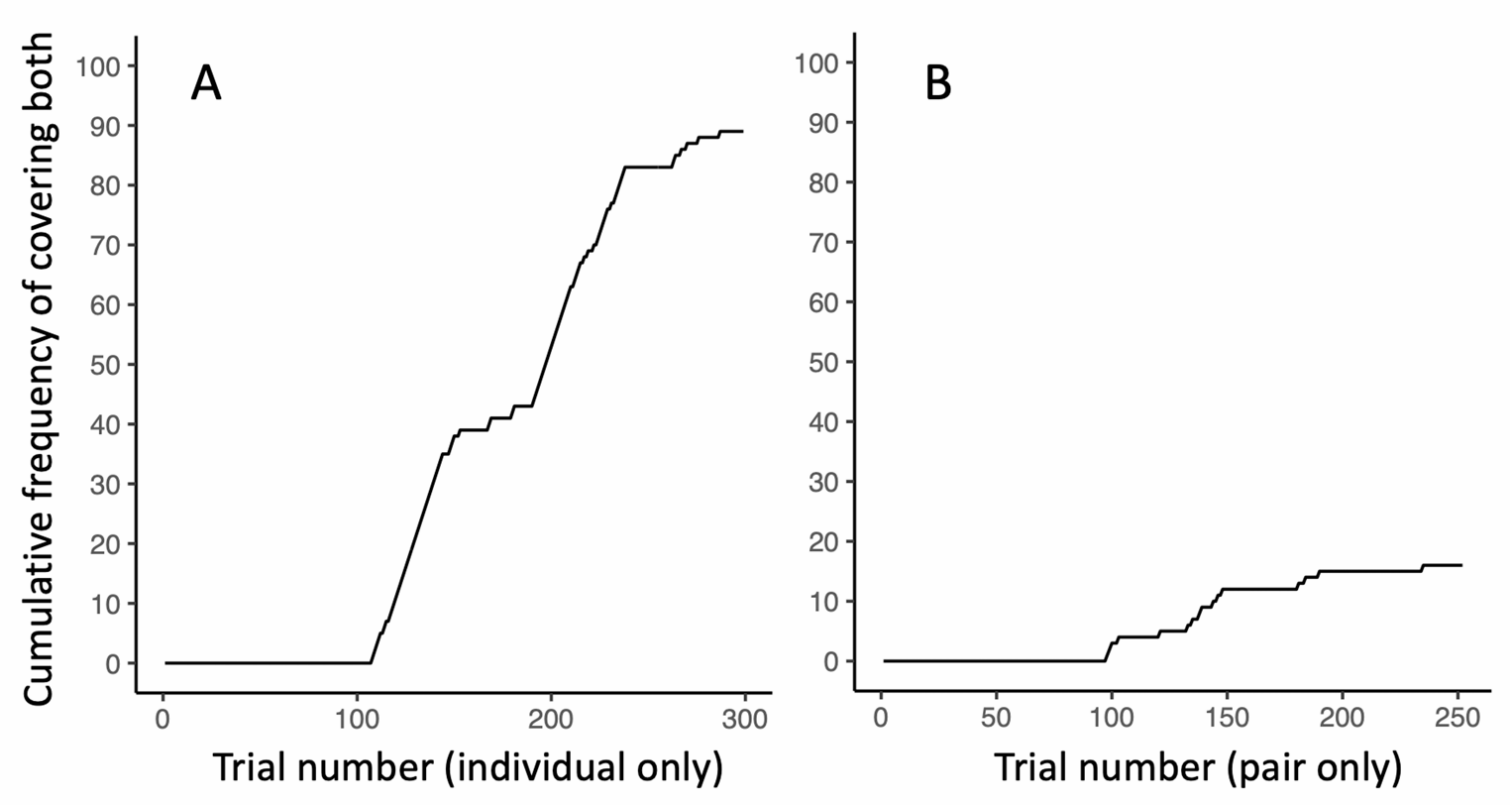
The cumulative frequency of Nammei covering both ends of the tube when tested (**A**) individually and (**B**) with her partner

We found that, although eight out of the twelve elephants covered both sides at least once (Table 1; Supplementary Material 1, Table S1), only one elephant, Nammei, relatively consistently covered both sides across trials (Supplementary Videos 2 and 3). She first covered both sides of the tube after 205 trials, during the ninth pair set, while her partner was not interacting with the tube. After this, when tested individually, she was relatively consistent in covering both sides (Figs. 2 and 3A), and covered both sides in 46.4% of her remaining individual trials (89 out of 192 trials; Supplementary Material 1, Table S1), although she sometimes reverted to covering one side (Fig. 3A). Furthermore, she was the only elephant who successfully ate the food reward at a rate greater than chance (percent success out of all individual trials subsequent to the first time she covered both: 118 out of 192 trials; 61.5%; *p* = 0.001; Supplementary Material 1, Table S1).

### Covering both sides of the tube collectively

Overall, both sides of the forked tube were covered in 35.0% (403 out of 1153) of pair trials. When pairs were analyzed separately, the maximum rate of covering both sides that a pair attained was 57.2% (103 out of 180 trials; Table 2). Across all pair trials, the food was eaten by either elephant in 60.1% (693 out of 1153) of trials, which is significantly greater than the success individuals could attain by chance (i.e., 50%; test of proportions: *p* < 0.001). When pairs were analyzed separately, we found that three out of the six pairs were successful at a rate significantly greater than 50% (Table 2) and two of those pairs were successful at a rate significantly greater than that which Nammei attained when she was tested individually (i.e., 61.5%; Tables 2 and 3). However, the success rate of Nammei and Pachee while in a pair was not greater than that of Nammei when tested individually (Tables 2 and 3). In addition, all pairs covered the same side of the tube at least once, and one pair exhibited this behavior in 16.6% (30 out of 181 trials) of trials (Table 2). Furthermore, at least one elephant within a pair covered the side farthest from where they were standing in 24.1% (287 out of 1153) of trials (Table 2).

**Table 2:** Summary of collective outcomes for all trials conducted in pairs

Pair	Total number of pair trials	Trials both sides covered	Trials only one side covered	Trials covering same	Trials covering opposite^a^	Trials food eaten (percent success)	*P*-value (test of proportions for 50%)^b^	*P*-value (test of proportions for 61.5%)^c^
Nammei and Pachee	252	95 (37.7%)	157 (62.3%)	5 (2.0%)	43 (17.1%)	159 (63.1%)	**< 0.001**	0.32
Phumpuong and Khod	180	55 (30.6%)	125 (69.4%)	9 (5.0%)	47 (26.1%)	97 (53.9%)	0.17	–
Alina and Malini	181	39 (21.5%)	142 (78.5%)	30 (16.6%)	69 (38.1%)	87 (48.1%)	0.67	–
Lawan and Wandee	180	24 (13.3%)	156 (86.7%)	1 (0.6%)	58 (32.2%)	91 (50.6%)	0.47	–
Malee and Sanlan	180	103 (57.2%)	77 (42.8%)	6 (3.3%)	29 (16.1%)	134 (74.4%)	**< 0.001**	**< 0.001**
Somsri and Baiboon	180	87 (48.3%)	93 (51.7%)	8 (4.4%)	32 (17.7%)	125 (69.4%)	**< 0.001**	**0.02**

The outcomes reported include the number (and percentage) of trials in which: (1) both sides of the forked tube were covered (i.e., collectively accounting for both sides from which the food could exit), (2) only one side was covered, (3) both elephants covered the same side, (4) at least one elephant covered the side farthest from the side on which they were standing (i.e., opposite), and (5) the food was eaten by either elephant (i.e., success). The results of tests of proportions are also reported to determine whether percent success was significantly greater than that which an individual could attain by chance (i.e., 50%) or that which Nammei attained individually after learning to cover both sides (i.e., 61.5%). Bold values indicate statistical significance

^a^Defined as either or both elephants covering the side that was farthest from where they were standing (including when one covered both sides)

^b^Alternative hypothesis: the proportion is greater than 0.5

^c^Alternative hypothesis: the proportion is greater than 0.615, which is the proportion of trials in which Nammei ate the food reward when tested individually (after the first time she covered both). This test was only conducted for pairs that had attained a percent success significantly greater than 50%

**Table 3:** Summary of Nammei’s behaviors when tested individually and with her partner, in trials before and subsequent to the first time she covered both sides of the forked tube

	When individual	When in pairs on right side—partner *not* interacting	When in pairs on left side—partner *not* interacting	When in pairs on right side—partner interacting	When in pairs on left side—partner interacting
	*Before the first time she covered both*				
Number of times Nammei ate food	49 out of 107 (45.8%)	11 out of 28 (39.3%)	10 out of 27 (37.0%)	2 out of 9 (22.2%)	12 out of 33 (36.4%)
	*After the first time she covered both*				
Number of times Nammei covered both	89 out of 192 (46.4%)	14 out of 39 (35.9%)	0 out of 24 (0.0%)	1 out of 55 (1.8%)	0 out of 36 (0.0%)
Number of times Nammei tried to scoop but got blocked by partner	NA	0 out of 39 (0.0%)	0 out of 24 (0.0%)	22 out of 55 (40.0%)	0 out of 36 (0.0%)
Number of times Nammei ate food	118 out of 192 (61.5%)	24 out of 39 (61.5%)	11 out of 24 (45.8%)	15 out of 55 (27.3%)	16 out of 36 (44.4%)
***P***-value (test of proportions)	**0.001**	0.10	0.58	> 0.99	0.69

The behaviors when Nammei was tested with a partner are separated by whether she was standing on the left or right side of the forked tube and by whether or not her partner was interacting with the tube. Covering both sides should have been easier for Nammei when she was standing on the right side of the tube—because she could place the tip of her trunk in the right opening and her trunk naturally scooped to the left—and when her partner was not interacting with the tube. The results of tests of proportions are reported to determine whether Nammei’s percent success was significantly greater than that which an individual could attain by chance (i.e., 50%). Bold values indicate statistical significance. Note that these tests examined only Nammei’s individual success and do not refer to collective success with her partner. All sides (right or left) are coded from the experimenter’s perspective

### Nammei’s behavior when tested in a pair

Nammei covered both sides significantly fewer times when she was tested with her partner, Pachee, compared to when she was tested individually (*X*^*2*^ = 74.6; *p* < 0.001; Table 3; Fig. 3B). However, Nammei still continued to attempt to cover both sides when she was with her partner. When in pairs, Nammei covered both sides in 10.3% of trials (16 out of 155 paired trials occurring after, and including, the first time she covered both). Like the first time she covered both sides, which occurred during a pair trial, she almost exclusively covered both sides when her partner was not interacting with the tube (Table 3). However, she *attempted* to cover both sides but was blocked by Pachee in 14.3% (22 out of 154) of trials subsequent to the first time she covered both (Table 3; Supplementary Material 1, Fig. S2). Nammei exclusively covered (or attempted to cover) both sides when she was standing on the right side (Table 3), likely because that was the side from which she was able to put her trunk tip in the right hole and scoop to the left (Fig. 2). However, even when Nammei was standing on the right side and Pachee was not interacting, Nammei still did not eat the food at a rate greater than chance (Table 3; *p* = 0.10).

### Switch vs. stay strategy

**Fig. 4:**
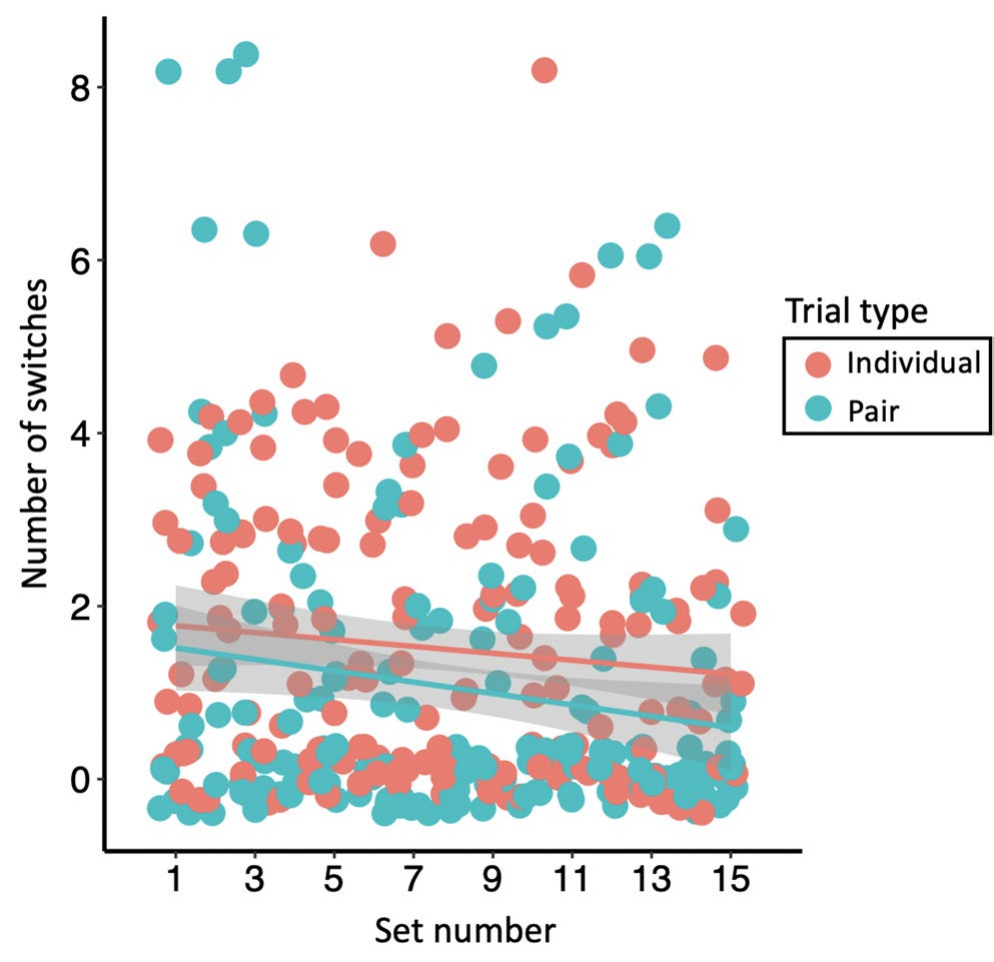
The number of times that elephants switched sides within a set decreased across sets (*X*^*2*^ = 14.8, *p* < 0.001) and was lower when elephants were tested in pairs compared to individually (*X*^*2*^ = 13.6, *p* < 0.001). Each point represents the number of switches—or instances in which they changed the side they covered—that a single elephant made in a single set (i.e., 12 trials). Each elephant participated in 15 individual and 15 pair sets. The shaded areas represent the 95% confidence intervals of the regression lines

Elephants varied in whether they switched which side they covered within a set, or if they always covered the same side (Supplementary Material 1, Table S2). However, the number of times that elephants switched sides within a set significantly decreased over time (*X*^*2*^ = 14.8, *p* < 0.001; Fig. 4), and was consistently significantly lower when elephants were tested in pairs compared to when they were tested individually (*X*^*2*^ = 13.6, *p* < 0.001; Fig. 4). On average, elephants switched sides 1.6 (±1.7 SD) times when tested individually and 1.0 (±1.8) time when tested in pairs, and all elephants stayed on the same side during at least one full set individually and one full set in pairs (Supplementary Material 1, Table S2).

### Inter-rater reliability and methodology verification

S.D. and the independent coder had almost perfect reliability (McHugh 2012) for identifying the side from which the food was dropped (Kappa = 0.989, *z* = 30.0, *p* < 0.0001), the side the elephant covered (Kappa = 0.959, *z* = 40.6, *p* < 0.0001), and whether the elephant ate the food (Kappa = 0.974, *z* = 29.6, *p* < 0.0001), and strong reliability for whether the elephant covered both sides (Kappa = 0.854, *z* = 26.0, *p* < 0.0001). When coding all of Nammei’s trials, S.D. and S.F.H. had almost perfect reliability for identifying the side from which the food was dropped (Kappa = 0.993, *z* = 23.3, *p* < 0.0001), the side the elephant covered (Kappa = 0.964, *z* = 34.9, *p* < 0.0001), whether the elephant covered both sides (Kappa = 0.931, *z* = 21.9, *p* < 0.0001), and whether the elephant ate the food (Kappa = 0.989, *z* = 23.2, *p* < 0.0001). When examining the additional endpoints that we coded concerning Nammei’s behavior in pairs, S.D. and S.F.H. had perfect reliability for whether Pachee was interacting with the tube (Kappa = 1.0, *z* = 7.75, *p* < 0.001), and strong reliability for whether Nammei attempted to cover both sides but was unable to because Pachee pushed her trunk out of the way (Kappa = 0.829, *z* = 5.75, *p* < 0.001).

We found that our methods worked as expected, with no evidence that elephants were able to use external cues to predict from which side the food would fall out. When elephants covered either the left or the right side, they chose the correct side in 49.9% (1869 out of 3749) of trials, which did not deviate from chance levels (test of proportions: *X*^*2*^ = 0.03, *p* = 0.56). Furthermore, the rate at which elephants ate the food reward when they only covered one opening was not greater than 50% (percent success: 45.0%; 1688 out of 3749 trials; test of proportions: *X*^*2*^ = 36.9, *p* > 0.99) suggesting that, despite the few instances (11 total trials) where elephants covered the incorrect opening but the food bounced off of the ramp and into reach of the elephant, elephants were not rewarded at a rate greater than chance.

## Discussion

Here, we did not find compelling evidence for Asian elephants’ ability to plan for mutually-exclusive outcomes either individually or collectively; however, we did observe some interesting behaviors that are worthy of discussion. When elephants were tested individually, no elephant quickly and consistently covered both sides of the forked tube without reverting back to covering one side—which is typically deemed as evidence for understanding mutually-exclusive outcomes through insight (Redshaw and Suddendorf 2016). However, after 205 trials, one elephant—Nammei—spontaneously covered both sides of the tube and then performed this behavior relatively consistently to obtain the food reward at a rate greater than chance. Although she needed much experience before performing this behavior and sometimes reverted to covering one side, we think that her performance is notable, but remains difficult to interpret in the context of understanding mutually-exclusive outcomes. Seven other elephants covered both sides at least once, and one caught food in the scoop of her trunk twice (Table 1; Supplementary Material 1, Table S1); however, none of these individuals exhibited this behavior consistently. When elephants were tested in pairs, although they performed significantly better (60.1% success, on average) than individuals could by chance (i.e., 50%), they were not close to achieving complete coordination (i.e., 100% success). This provides some evidence for the most basic form of collective intelligence—where the collective simply outperforms the individual (Krause et al. 2010; Green 2015)—but is weak evidence for collective planning for mutually-exclusive outcomes because pairs only accounted for both possibilities (i.e., both sides of the tube covered) in 35.0% (403 out of 1153) of trials. Therefore, although we did not find conclusive evidence for individual or collective preparation for mutually-exclusive future outcomes, our results suggest that this remains a fruitful avenue for future research and that more studies using alternative experimental paradigms are likely needed to further elucidate elephants’ capacity for future planning.

### Individually preparing for mutually-exclusive events

When tested individually on the forked tube task, elephants performed similarly to 2 year-old human children and some non-human primates. Researchers who have used the forked tube paradigm have shown that 4 year-old children cover both sides of the tube in the first trial and then cover both sides consistently, suggesting that they use insight to prepare for mutually-exclusive outcomes (Redshaw and Suddendorf 2016; Suddendorf et al. 2017; Redshaw et al. 2019). On the other hand, 2 year-old children rarely cover both sides on the first trial, but some can learn to do so after experiencing multiple trials; following this, some individuals continue to cover both sides consistently and others revert back to only covering one side. Researchers have interpreted this behavior to mean that these younger children can learn the contingencies of the task through trial-and-error, but may not plan cognitively for the mutually-exclusive outcomes (Redshaw and Suddendorf 2016; Suddendorf et al. 2017, 2020; Redshaw et al. 2018, 2019). Some studies have shown that, similar to human children below the age of 4, non-human primates do not cover both sides on the first trial, but that certain individuals of certain species learn to cover both sides of the forked tube relatively consistently (Redshaw and Suddendorf 2016; Suddendorf et al. 2017, 2020; Lambert and Osvath 2018). For example, Redshaw and Suddendorf (2016) found that, although three chimpanzees (*Pan troglodytes*) and five orangutans (*Pongo abelii*) did not cover both sides of a forked tube on their first trials, two chimpanzees and one orangutan covered both sides in at least one trial, and one chimpanzee covered both sides in 34 out of 84 trials, with a maximum of 13 consecutive trials. Interestingly, in a recent study, Warren et al. (unpublished data) found that four chimpanzees (out of 21) covered both sides on their first trial of a forked tube task, and that three individuals covered both sides in 18 out of 18 consecutive trials, suggesting that chimpanzees can perform similarly to 4-year-old children and spontaneously prepare for mutually-exclusive outcomes. In the current study, Nammei learned to cover both sides after experiencing multiple trials and, when tested individually, covered both sides in 89 out of 299 trials with a maximum of 28 consecutive trials (Fig. 3A). Therefore, although we do not exclude the possibility that Nammei learned the need to account for both outcomes after much experience with the forked tube, our results are more consistent with the interpretation that she simply learned through trial-and-error that positioning her trunk in a scooping motion allowed her to gain the food reward more often than chance. Previous studies have shown that Asian elephants can solve novel problems through trial-and-error learning or innovation (Barrett and Benson-Amram 2021; Jacobson et al. 2022, 2023), and the current study supports elephants’ capacity for learning when presented with unique foraging tasks.

The finding that only one elephant consistently covered both sides may reflect factors other than a limitation in the animals’ ability to account for multiple future outcomes through insight or trial-and-error learning. First, although elephants in captive settings are frequently observed positioning their trunk in a scooping motion when receiving food or water from human handlers and, thus, should be physically capable of performing this movement, it may nevertheless be a physically difficult and perhaps unintuitive behavioral response in this context. In non-human primates, there is evidence that using two hands simultaneously is also difficult (Lambert and Osvath 2018; Suddendorf et al. 2020), and may be an alternative explanation for why some individuals do not cover both sides in the forked tube task. Similarly, there is evidence that 3-year-old children only cover one side in this task not because they cannot understand mutually-exclusive outcomes, but because the act of covering both sides may be unintuitive; in one study where 3-year-olds were first shown both strategies (cover one side, cover both sides), they covered both sides in the forked tube task at a similar rate to 4-year-olds (Turan-Küçük and Kibbe 2024). In the future, researchers may consider training elephants on the scooping strategy before testing them with the forked tube. Furthermore, and in contrast to studies with two-handed individuals, even when elephants performed the scooping motion, success was not guaranteed. Indeed, even Nammei only caught the food when it fell out of the side opposite to the tip of her trunk (i.e., in the ‘scoop’ of her trunk) 47.6% of the time (20 out of 42 trials in which she covered both and the food fell from the side opposite to the tip of her trunk; Supplementary Material 1, Table S1). Therefore, some elephants may not have been motivated to cover both sides. For example, even if elephants understood the mutually-exclusive outcomes of the task, understood that a scooping behavior could be a reliable solution, and could physically manipulate their trunk to cover both sides, they were faced with two choices: (1) engage in a trunk behavior that is potentially difficult and get a small piece of apple up to 100% of the time or (2) easily cover a single side and get a small piece of apple 50% of the time. For a 3,000 kg mammal requiring up to 250 kg of food per day, the difference between six and twelve small pieces of apple per set may be negligible. Thus, this makes the finding that Nammei relatively consistently covered both sides—despite potential challenges and low pay-off—nevertheless notable.

Nammei may have been the only elephant to exhibit this behavior because she had a behavioral predisposition. Specifically, even when she covered only one side, she naturally scooped her trunk. In earlier trials, she almost always covered the left side, where she scooped her trunk but the ‘scoop’ was not underneath the tube. So, to cover both sides, she only needed to put the tip of her trunk under the right side to then have the ‘scoop’ in the correct position. Interestingly, she covered both sides for the first time when she was in a pair trial but her partner was not interacting with the tube. This could have led to her covering both sides in two ways. First, innovation may be fostered by competition (Brosnan and Hopper 2014). Indeed, Warren et al. (unpublished data) found that, when chimpanzees were tested on the forked tube task first individually (‘pre-pair’), then in pairs, and then again individually (‘post-pair’), individual chimpanzees covered both sides (i.e., using two hands) at a higher frequency in pair and post-pair trials, compared to pre-pair trials. This suggests that the social context may have facilitated the use of the two-handed ‘cover both’ behavior. On the other hand, having a partner there may have simply forced Nammei to stand on the right side of the tube, so she put the tip of her trunk under the right side instead of the left (her usual side bias). We also cannot discount the possibility that the methodological decision to rub food on the bottoms of both openings of the tube (to maintain elephants’ interest) may have provided two non-mutually exclusive cues to the elephants (i.e., persistent odor emanating from each side simultaneously). Thus, Nammei may have covered both openings of the forked tube simply because she was responding to these cues. However, we think it is unlikely that this was a strong driver of behavior because, if so, we would have expected to see frequent and consistent covering of both openings across elephants, which was not the case.

Because there are multiple reasons why elephants may not have covered both sides consistently, we also examined their behavior further by investigating switch vs. stay strategies. We found that, in earlier sets, elephants tested individually switched sides often—suggesting that they were trying to predict from which side the food would fall. Then, in later sets, elephants switched sides less frequently. This could indicate that they learned the contingencies of the task over time because, if they understood that the food would randomly fall from either side in each trial but they either (1) were not motivated to work harder to be successful in 100% of trials compared to 50%, (2) could not think of a way to use their trunk to cover both sides, or (3) had a physically difficult time covering both sides, then their best strategy would be to always cover the same side. Thus, elephants who did not cover both ends may have shown some evidence that they understood that the food had an equal possibility of coming out of either side. Alternatively, consistently covering one side as trials progressed may suggest that the elephants first attempted to predict the outcome but, when no reliable solution emerged, shifted to choosing the side that they believed most often yielded a reward (Redshaw and Suddendorf 2020). Non-human primates vary in whether they cover the same side or switch sides in the forked tube task (Redshaw and Suddendorf 2016; Suddendorf et al. 2017; Lambert and Osvath 2018), although how this behavior changes with experience was not investigated in these studies.

A next step to continue to investigate whether elephants can plan for mutually-exclusive events would be to use a different type of task. For example, Engelmann et al. (2023) used a paradigm where chimpanzees had to stabilize trays, which could be physically easier than using both hands to catch a reward (but see Redshaw and Suddendorf 2024, in which concerns are presented about the rationale, results, and interpretation of this study). Chimpanzees were presented with a forked tube that was positioned above two trays containing food. Individuals had to stabilize the trays so that, when a rock was dropped down the tube, it would not tilt backward and fall toward a human competitor. Chimpanzees were more likely to stabilize both trays when the rock fell out of the forked tube—where it was uncertain on which tray the rock would fall—than when it fell out of a straight tube—where it was certain on which tray the rock would fall. This suggests that they understood and prepared for both possible outcomes when the future was uncertain. In addition, Engelmann et al. (2021) showed that, when chimpanzees were given the opportunity to pull two ropes to access containers with food inside them, they pulled both ropes more often when the location of the food was uncertain (i.e., hidden in one of two opaque boxes) compared to when the location was certain (i.e., either placed in one of two transparent boxes or placed in an opaque box while the subject could observe). This suggests that chimpanzees consider alternative possibilities and understand that the food could be in either container when the context is uncertain. Interestingly, this study differs from forked tube studies because chimpanzees must consider alternatives of an event that already occurred (i.e., epistemic uncertainty; Robinson et al. 2006), whereas in the forked tube paradigm, animals must consider alternatives of an event that has not yet occurred (i.e., physical uncertainty; Robinson et al. 2006). In the future, researchers could consider using methods that are physically easier for elephants to perform, and may also consider testing this question in a framework about epistemic uncertainty rather than physical uncertainty (Robinson et al. 2006).

### Collectively preparing for mutually-exclusive events

Collective intelligence can be defined as when group performance is greater than individual performance, regardless of the cognitive mechanisms that may underlie such behavior or the understanding of the complexity of the task by the individuals involved (Pratt 2009; Theiner et al. 2010; Green 2015). Although the results we found when elephants were tested in pairs do fall within the definition of collective intelligence—i.e., pairs performed significantly better (60.1% success, on average) than individuals could by chance (i.e., 50%)—we find it difficult to interpret these results. When pairs were analyzed separately (Table 2), three out of six pairs performed better than individuals could by chance, and two pairs (Malee with Sanlan and Somsri with Baiboon) performed better than an elephant who covered both sides when tested individually (i.e., Nammei, 61.5%). No pair attained a success rate of 100%, which would have been possible if pairs had perfectly coordinated their behavior to account for both mutually-exclusive outcomes (i.e., cover both sides). Indeed, coordination was far from perfect, with even the pair with the greatest coordination only covering both sides of the tube in 57.2% (103 out of 180) of trials (Table 2). Warren et al. (unpublished data) also found that pairs of chimpanzees rarely coordinated their behavior to cover both sides of a forked tube; indeed their performance appeared to be worse than elephants’, where pairs of chimpanzees coordinated to each cover one side in only 12 out of 684 total trials. In our study, it is possible that a percent coordination of 100% was not the ideal benchmark with which to compare elephant pair performance. In the future, researchers may consider conducting a control condition where the outcome is certain (i.e., pairs know from which side the food will fall). This would allow for a comparison between how often pairs cover both sides in an ‘uncertain’ versus a ‘certain’ condition, instead of simply comparing percent coordination to 100%. Nevertheless, it is possible that elephants may not display more than the most basic form of collective intelligence in the context of immediate food acquisition because they are herbivorous browsers and grazers (Sukumar 1990) and may not need to act collectively in a foraging context in the wild, where their primary food sources are not easily monopolized. Instead, they may be more likely to act collectively for other reasons, such as when navigating to distant resources or protecting their young calves.

Although the phenomenon of collective intelligence in a group does not necessitate that individuals understand the task (Theiner et al. 2010; Camazine et al. 2020), certain behaviors that elephants exhibited in pairs may shed light on how much individuals understood about the contingencies of the task. For example, if individual elephants understood that there was an equal probability of the food coming out of either side of the tube, we might expect them to understand that it is counterproductive to compete for a specific side of the tube when in pairs. In contrast, if elephants did not understand the contingencies of the task and were instead attempting to predict from which side the food would come out, then they may think that they should compete for a specific side. Here, we found that elephants occasionally tried to cover the same side (Supplementary Material 1, Fig. S1; Supplementary Video 4), and the side that was farthest away from them (i.e., ‘covered opposite’; Table 2). This may suggest that they were attempting to predict from which side the food would come out, or simply attempting to ensure that their partner did not get the food, instead of working as part of a collective. Accordingly, pairs that covered the same and opposite side the most frequently were the pairs that had the lowest collective success (Table 2). It is important to note that the context of this experiment was not cooperative because the food reward was not shareable. Therefore, elephants may have been less inclined to reflect on the contingencies of the task and more inclined to compete. Indeed, when Li et al. (2021) tested Asian elephants who were free to choose their partner in a cooperative problem-solving task, they found that elephants cooperated and mitigated competition when the food reward was shareable, but that cooperation broke down when food was not shareable. However, it is also possible that elephants in the current study attempted to mitigate competition and improve coordination. For example, elephants switched sides within a set fewer times overall when they were in pairs compared to when tested individually, and elephants switched sides within a set fewer times in later compared to earlier sets. This suggests that pairs may be attempting to coordinate their behavior to cover both sides—each consistently choosing their respective side—and that, over time, they are learning to better coordinate. Indeed, collectives can learn over time (reviewed in Biro et al. 2016). For example, pairs of homing pigeons (*Columba livia*) can learn to take more efficient travel routes over time (Pettit et al. 2013), and colonies of *Leptothorax albipennis* ants become more efficient at moving to new nesting sites after repeated emigrations (Langridge et al. 2004). It is possible that pairs of elephants, too, became more efficient at coordinating their movements to each cover their respective sides after repeated trials (Supplementary Video 5). In addition, the behavior of ‘cover opposite’ could potentially be a social behavior that indicates trust between partners (i.e., showing mutual trust for putting trunks close to one another), instead of a competitive behavior; it is important to note that, if performed simultaneously by each elephant, this behavior would still result in collective success.

Because Nammei could successfully monopolize the tube to cover both sides—and thus was the only individual who could have truly competed with her partner—we examined her behavior with her partner more closely. We aimed to determine whether she (1) tried to monopolize the tube because she knew how to cover both sides or (2) reverted to covering one side to act as part of a collective and coordinate behavior with her partner. After the first time she covered both, Nammei covered both sides fewer times when she was with her partner (15 out of 154 trials; 9.7%) compared to when she was tested individually (89 out of 192 trials; 46.4%). Furthermore, out of the paired trials in which she successfully covered both sides, the majority of instances (14 out of 15 trials) were when her partner was not interacting with the apparatus (Table 3). This could suggest that she was coordinating her behavior to be part of a collective. However, we also noted 22 instances when Nammei attempted to cover both sides, but she could not because her partner’s trunk blocked her (Table 3; Supplementary Material 1, Fig. S2). If we count these instances as instances in which Nammei covers both, then she covered both sides 37 times (out of 154 trials; 24.0%, starting after the first time she covered both) when with her partner (Table 3). Although this is still lower than the amount of times she covered both when tested individually, it suggests that she was not consistently reverting to covering one side to coordinate with her partner, but was instead likely attempting to monopolize the tube.

## Conclusions

After much experience, one Asian elephant learned to relatively consistently cover both sides of a forked tube to obtain a food reward at a rate greater than chance. However, overall, elephants performed similarly to 2 year-old children and some non-human primates—who can sometimes produce the solution to maximize their success in this task through trial-and-error learning. Nonetheless, it is noteworthy that *even one* elephant covered both sides of the tube consistently, considering the potentially difficult trunk ‘scooping’ motion that was necessary. Importantly, we do not discount the possibility that elephants understood that the outcomes of this task were mutually-exclusive, but simply did not understand how to—or were not motivated to—account for both possibilities. In pairs, elephants did not collectively maximize success, but the non-sharable aspect of this study may have made the context competitive, resulting in competition between elephants rather than coordination. Although this study does not provide strong evidence for future planning or collective intelligence beyond its most basic form, more research on these topics in elephants is warranted. In the future, questions about future planning, collective intelligence and the understanding of mutually-exclusive outcomes could be examined using a different paradigm and/or in a cooperative context, which may be more salient and thus more ecologically valid for elephants.

## Supplementary Information

Below is the link to the electronic supplementary material.

Supplementary Material 1

Supplementary Video 1

Supplementary Video 2

Supplementary Video 3

Supplementary Video 4

Supplementary Video 5

## Data Availability

The data that support the findings of this study are available on Figshare: 10.6084/m9.figshare.29323367.v1.
