# Supplementary material for: Do Asian elephants plan for mutually-exclusive outcomes?
Source: Anim Cogn. 2025 Nov 12;28(1):93. doi: 10.1007/s10071-025-02009-1 (PMC12612009; doi:10.1007/s10071-025-02009-1)
Supplement: Supplementary file 1 — Supplementary Material 1 [file 10071_2025_2009_MOESM1_ESM.pdf]

SUPPLEMENTARY INFORMATION

Title: Do Asian elephants plan for mutually-exclusive outcomes?

Author List: Sydney F. Hope[1*], Sangpa Dittakul[2], Marnoch Yindee[3], Taweepoke Angkawanish[4], Joshua M. Plotnik[1,5*]

1. Department of Psychology, Hunter College, City University of New York, New York, USA
2. Golden Triangle Asian Elephant Foundation, Chiang Saen, Chiang Rai, Thailand
3. Akkhraratchakumari Veterinary College, Walailak University, Nakhon Si Thammarat, Thailand
4. National Elephant Institute, Forest Industry Organization, Lampang, Thailand
5. Psychology Ph.D. Program, CUNY Graduate Center, City University of New York, New York, USA

**Corresponding authors:* SFH – sydney.hope@gmail.com, ORCID 0000-0002-3711-8593;

JMP – joshua.plotnik@gmail.com, ORCID 0000-0002-7597-8818

Additional behavioral details for all elephants who covered both sides at least once

Table S1. Summary of behaviors of all elephants who covered both sides at least once. The trial number and type (individual or pair) in which each elephant first covered both and the strategy they used (i.e., in which hole they put their trunk and in which direction they scooped the elbow of their trunk) are stated. All sides (right or left) are coded from the experimenter's perspective. The number (and percentage) of trials in which each elephant covered both sides and ate the food are reported for both individual and pair trials. The subsets of trials used to calculate the percentages are described in the parentheses in the contents of the first column. *P*-values are reported for tests of proportions to determine whether the proportion of trials in which elephants ate the food (i.e., success) was significantly greater than 50%. Note that, even for pair trials, percent success is based on the success of the individual (i.e., not collective success). Bolded values indicate significance.

	**Elephant name**							
	**Nammei**	**Pachee**	**Phumpuong**	**Khod**	**Alina**	**Lawan**	**Malee**	**Wandee**
First trial cover both (out of all trials)	205 out of 551	101 out of 433	32 out of 360	1[a] out of 357	216 out of 361	2 out of 360	134 out of 360	41 out of 360
Type of trial where first cover both	pair	individual	individual	individual	individual	pair	individual	individual
Strategy	Trunk tip in right hole and scoop to cover left	Trunk tip in left hole and scoop to cover right	Trunk tip in right hole and scoop to cover left	Trunk tip in left hole and scoop to cover right	Trunk tip in right hole and scoop to cover left	Trunk tip in left hole and scoop to cover right	Trunk tip in left hole and scoop to cover right	Trunk tip in right hole and scoop to cover left
	Individual trials							
Number of times covered both (% out of all individual trials, starting *after* first time covered both)	89 out of 192 (46.4%)	0 out of 116 (0.0%)	7 out of 148 (4.7%)	2 out of 176 (1.1%)	0 out of 73 (0.0%)	0 out of 180 (0.0%)	0 out of 118 (0.0%)	1 out of 175 (0.6%)
Ate food when they covered both (% out of all trials covered both)	67 out of 89 (75.3%)	0	3 out of 8 (37.5%)	2 out of 3 (66.7%)	0	NA	0	1 out of 2 (50.0%)
Ate when food fell from opposite side of trunk tip (% out of all trials covered both and food fell from opposite side)	20 out of 42 (47.6%)	0	0 out of 5 (0.0%)	2 out of 2 (100%)	0	NA	0	0 out of 1 (0.0%)
Percent success (eat) (% out of all individual trials, starting after first time covered both)	118 out of 192 **(61.5%)**	52 out of 116 (44.8%)	66 out of 148 (44.6%)	85 out of 176 (48.3%)	30 out of 73 (41.1%)	89 out of 180 (49.4%)	58 out of 118 (49.2%)	77 out of 175 (44.0%)

P-value (test of proportions for percent success)	**0.001**	0.85	0.89	0.65	0.92	0.53	0.54	0.93
				Pair trials				
Number of times covered both (% out of all pair trials, starting *after* first time covered both)	15 out of 154 (9.7%)	0 out of 216 (0.0%)	1 out of 180 (0.6%)	0 out of 180 (0.0%)	0 out of 73 (0.0%)	1 out of 178 (0.6%)	0 out of 108 (0.0%)	0 out of 144 (0/0%)
Ate food when they covered both (% out of all trials covered both)	10 out of 16 (62.5%)	NA	0 out of 180 (0.0%)	NA	NA	0	NA	NA
Ate when food fell from opposite side of trunk tip (% out of all trials covered both and food fell from opposite side)	6 out of 8 (75.0%)	NA	NA[b]	NA	NA	0	NA	NA
Percent success (eat) (% out of all pair trials, starting after first time covered both)	66 out of 154 (42.9%)	55 out of 216 (25.5%)	42 out of 180 (23.3%)	54 out of 180 (30.0%)	17 out of 72 (23.6%)	82 out of 178 (46.1%)	41 out of 108 (38.0%)	5 out of 144 (3.5%)
P-value (test of proportions for percent success)	0.95	>0.99	>0.99	>0.99	>0.99	0.84	0.99	>0.99

[a]Before this, Khod underwent six trials that were part of three incomplete sets (due to disinterest) which were excluded, so this was not her very first interaction with the forked tube.

[b]In this single trial, Phumpuong put her trunk under the left side but then quickly switched to the right side as the food dropped. This was coded as 'cover both' for analyses, but she did not scoop her trunk. This was the only instance in which an elephant performed a behavior like this.

Summary statistics for switching behavior

Table S2. Summary statistics (mean, standard deviation, minimum, and maximum) of the number of times in which elephants switched sides within sets, both individually and in pairs. If, for example, within a 12-trial set, an elephant chose to cover the left side five times, both sides one time, and then the right side six times, they would receive a ‘2’ because they switched their choice two times (i.e., from left to both, and then from both to right). In contrast, if an elephant consistently chose to cover the same side, they would receive a value of ‘0’.

	Individually				Pairs			
**Name**	**Mean**	**SD**	**Min**	**Max**	**Mean**	**SD**	**Min**	**Max**
Alina	2.7	1.6	0	4	2.1	1.6	0	4
Baiboon	0.4	0.7	0	2	0.5	1.0	0	3
Khod	2.4	2.1	0	8	3.0	3.3	0	8
Lawan	0.5	0.6	0	2	0.7	0.9	0	3
Malee	1.3	1.2	0	3	0.7	1.0	0	3
Malini	0.8	1.1	0	3	0.9	1.6	0	5
Nammei	1.6	2.1	0	7	1.6	2.4	0	6
Pachee	2.2	2.1	0	6	0.3	0.8	0	3
Phumpuong	1.9	1.7	0	5	0.9	1.6	0	6
Sanlan	1.0	1.3	0	4	0.7	1.0	0	3
Somsri	1.3	1.7	0	4	0.9	1.7	0	6
Wandee	2.5	1.7	0	6	0.3	1.0	0	4

Behaviors of interest during pair trials

1) Covering the same side

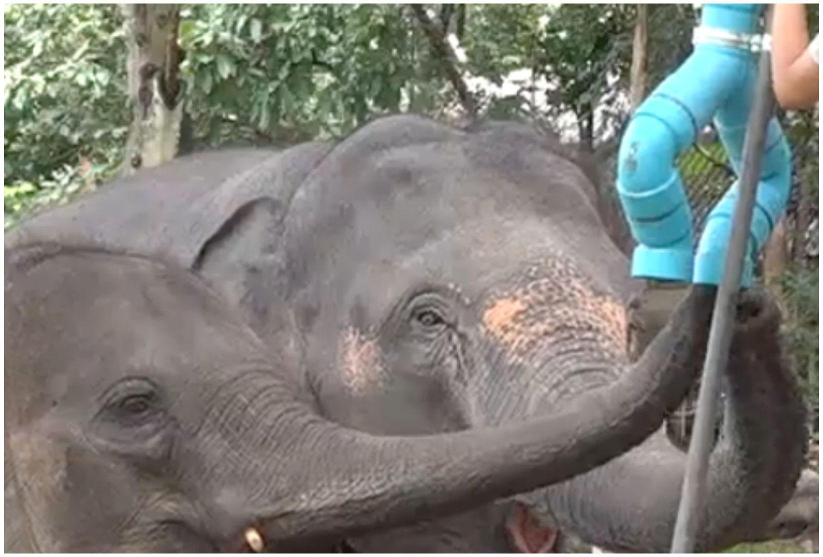

Figure S1. Baiboon and Somsri each put their trunk underneath the same side of the tube. This behavior potentially shows a non-understanding of the task. Since the food has an equal probability of coming out of either side, an elephant who understands the task should know that it is not beneficial for both elephants to cover the same side. If elephants do not understand the contingencies and attempt to predict from which side the food will fall, this behavior may be a competitive attempt to monopolize the food. Alternatively, if they do understand that there is an equal probability for the food to fall from either side, this behavior may be a way in which a competitive elephant ensures that their partner does not obtain the food, regardless of whether they get the food for themselves. Photo is a still image captured and cropped from a video taken with a Sony FDR-AX100.

2) Attempted scoop blocked by partner

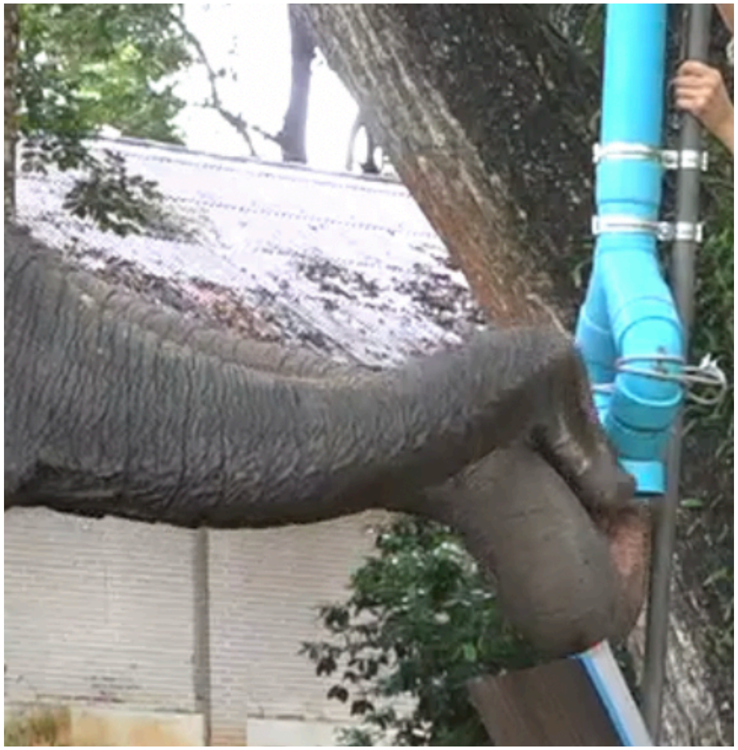

Figure S2. Nammei is attempting to cover both sides but is blocked by her partner, Pachee. Pachee's trunk is in the foreground and Nammei's trunk is behind it and performing the scooping motion. Pachee's trunk is reaching for the hole of the tube, so that Nammei is not able to cover both sides. Photo is a still image captured and cropped from a video taken with a Sony FDR-AX100.

Supplementary videos – descriptions

Video 1. An elephant (Wandee) covers one side of the tube. Two trials are shown here. In both trials, she covers the right side of the tube (from the experimenter's perspective). In the first trial, the food falls from the right side and in the second trial the food falls from the left side. Video was taken on a Sony FDR-AX100 camcorder and was cropped to exclude extraneous environmental information.

Video 2. Nammei performing the "cover both", or "scoop", behavior. The food falls out of the left opening of the tube; Nammei puts the tip of her trunk in the right opening and scoops her trunk to catch the food that falls from the left opening (from the viewer's perspective in the left video; from the experimenter's perspective in the right video). Two different camera angles of the same trial are presented here, combined using iMovie. Videos were taken on a GoPro HERO8 Black (left) and a GoPro HERO7 White (right) and were cropped to exclude extraneous environmental information.

Video 3. This is the same trial as in Video 2, but from two different angles (combined using iMovie). Videos were taken on two camcorders, a Sony FDR-AX100 (left) and a Sony HDR-CX405 (right), and were cropped to exclude extraneous environmental information. The color (highlights and shadows) was adjusted in the video on the right for clarity.

Video 4. Two elephants (Baiboon, left; Somsri, right) cover the same side of the tube when the food is dropped. The food drops from the opening that both elephants are attempting to cover. Notice that neither elephant catches the food, and it falls down the ramp and out of reach. Video was taken on a Sony FDR-AX100 camcorder and was cropped to exclude extraneous environmental information.

Video 5. A pair of elephants (Sanlan and Malee), where each elephant covers one side. Two trials are shown here. In the first trial, Sanlan (elephant with tusks) gets the food; in the second trial, Malee (without tusks) gets the food. Two different camera angles of the same trial are presented here, combined using iMovie. Videos were taken on Sony FDR-AX100 (left) and Sony HDR-CX405 (right) camcorders and were cropped to exclude extraneous environmental information.
